# Vortex Domain Wall Thermal Pinning and Depinning in a Constricted Magnetic Nanowire for Storage Memory Nanodevices

**DOI:** 10.3390/nano14181518

**Published:** 2024-09-19

**Authors:** Mohammed Al Bahri, Salim Al-Kamiyani, Al Maha Al Habsi

**Affiliations:** Department of Basic and Applied Sciences, A’Sharqiyah University, P.O. Box 42, Ibra 400, Oman

**Keywords:** micromagnetic simulation, vortex domain wall, stepped magnetic nanowire, spin transfer torque, VDW thermal stability

## Abstract

In this study, we investigate the thermal pinning and depinning behaviors of vortex domain walls (VDWs) in constricted magnetic nanowires, with a focus on potential applications in storage memory nanodevices. Using micromagnetic simulations and spin transfer torque, we examine the impacts of device temperature on VDW transformation into a transverse domain wall (TDW), mobility, and thermal strength pinning at the constricted area. We explore how thermal fluctuations influence the stability and mobility of domain walls within stepped nanowires. The thermal structural stability of VDWs and their pinning were investigated considering the effects of the stepped area depth (*d*) and its length (*λ*). Our findings indicate that the thermal stability of VDWs in magnetic stepped nanowires increases with decreasing the depth of the stepped area (*d*) and increasing nanowire thickness (*th*). For *th* ≥ 50 nm, the stability is maintained at temperatures ≥ 1200 K. In the stepped area, VDW thermal pinning strength increases with increasing *d* and decreasing *λ*. For values of *d* ≥ 100 nm, VDWs depin from the stepped area at temperatures ≥ 1000 K. Our results reveal that thermal effects significantly influence the pinning strength at constricted sites, impacting the overall performance and reliability of magnetic memory devices. These insights are crucial for optimizing the design and functionality of next-generation nanodevices. The stepped design offers numerous advantages, including simple fabrication using a single electron beam lithography exposure step on the resist. Additionally, adjusting *λ* and *d* allows for precise control over the pinning strength by modifying the dimensions of the stepped areas.

## 1. Introduction

The development of storage memory nanodevices has witnessed remarkable advancements in recent years, driven by the need for higher data storage densities and faster processing speeds [1,2,3,4,5]. A critical component of these devices is the magnetic nanowire, composed of ferromagnetic (FM) and antiferromagnetic (AFM) materials, which serve as the backbone for various memory technologies. The key difference between FM and AFM nanowires lies in the alignment of their magnetic moments. In FM nanowires, the magnetic moments align parallelly, resulting in a strong net magnetic moment, which enables them to retain magnetization and makes them ideal for data storage applications. In contrast, AFM nanowires have magnetic moments that align in opposite directions, canceling each other out, leading to no net magnetic moment. This makes AFM nanowires insensitive to external magnetic fields, reducing interference and enhancing data security in storage devices [6,7,8]. Within these nanowires, vortex domain walls (VDWs) play a pivotal role in determining the efficiency and reliability of data storage and retrieval processes, with their unique properties such as chirality, nanoscale size, pinning and depinning behavior, thermal stability, low critical currents, and compatibility with spintronic technologies, making them a powerful tool for advanced data storage devices. However, understanding the behavior of VDWs, particularly their thermal pinning and depinning characteristics, is crucial for optimizing the performance of magnetic nanowires in memory applications [9,10,11,12,13].

VDWs are formed due to the unique magnetic configurations within nanowires, and their stability is influenced by both intrinsic material properties and external factors such as temperature and current density. The phenomena of thermal pinning and depinning dictate the mobilization of these domain walls under varying thermal conditions. Understanding these processes is essential for enhancing the design and functionality of memory nanodevices [14,15,16,17,18]. Recent studies have highlighted the significance of constricted magnetic nanowire dimensions, which directly impact the thermal stability of VDWs. Experimental and theoretical investigations have shown that achieving precise control over domain wall propagation behavior is essential for these devices, and this can be accomplished by using local pinning centers that establish well-defined, stable wall positions. For instance, H. Y. Yuan and X. R. demonstrate that the presence of notches can effectively pin domain walls at specific locations within the nanowire. Kurniawan and Djuhana found that current-driven DW depinning in permalloy nanowires is sensitive to notch geometry, with higher current densities aiding depinning. Furthermore, Brandão et al. have shown that asymmetric notches can control magnetic vortex chirality [19,20,21,22,23,24].

Extensive research has been conducted on the thermal behavior of VDWs in magnetic nanowires. Some studies have explored the impact of current-induced domain wall motion, providing critical insights into the factors influencing VDW mobility. Additionally, other investigations have deepened our understanding of the thermal effects on domain wall behavior, emphasizing the importance of temperature control in device operation [25,26,27,28].

The interaction of VDWs with constrictions in magnetic nanowires has been a focal point of research, with several studies demonstrating the critical role of geometrical constraints in domain wall dynamics. These studies illustrate how engineered nanowire geometries can be utilized to control VDW movement, thereby enhancing the functionality of magnetic memory devices [29,30,31,32,33,34].

Theoretical models have also played a significant role in elucidating the mechanisms of VDW thermal pinning and depinning. Some works have provided comprehensive frameworks for understanding the energy barriers associated with domain wall motion, offering predictive tools for designing more efficient nanowire-based devices [35,36,37,38,39].

**Figure 1:**
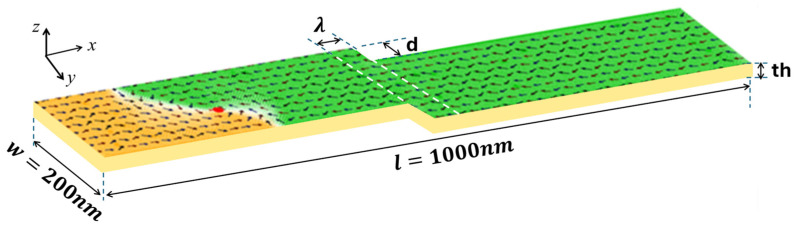
The stepped nanowire dimensions of 1000 nm in length and 200 nm in width with a VDW.

This article aims to consolidate current knowledge on VDW thermal pinning and depinning in constricted magnetic nanowires, providing a thorough overview of theoretical models. By examining the effects of step depth (*d*) and length (*λ*) [Figure 1] on VDW stability, we seek to offer insights into the optimization of nanowire designs for enhanced storage memory applications. This study leverages a broad array of references to present a comprehensive perspective on the topic, drawing from foundational research to the latest advancements in the field. Specifically, this study examines the thermal stability of VDWs by addressing three primary factors: (1) the thermal transformation of VDWs during dynamic motion, (2) the impact of device temperature on VDW behavior, and (3) the thermal pinning and depinning process of VDWs in the stepped region.

## 2. Theoretical Model

The Object Oriented Micro-Magnetic Framework (OOMMF) project [40] was employed to perform magnetic simulations, solving the Landau–Lifshitz–Gilbert (LLG) equation [41]. In cases where the current is aligned with the wire axis, the LLG equation is expressed as

(1) $$\frac{dm}{dt}=-\gamma m\times H_{eff}+H_{th}+\alpha m\times \frac{dm}{dt}-\left(u\cdot \nabla \right)m+\beta m\times \left(u\cdot \nabla \right)$$

where γ, α, $H_{eff}$, $H_{th}$, ∇, and m represent the gyromagnetic ratio, Gilbert damping parameter, effective magnetic field, thermal field, operator nabla, and unit vector of magnetization, respectively. The relationship between the thermal field and device temperature is described by the following equation:

(2) $$\langle H_{th,i}(r,t),H_{th,j}(\overset{´}{r},\overset{´}{t})\rangle =\frac{2\alpha k_{B}}{\gamma \mu_{0}M_{s}V}\delta_{ij}\delta (r-\overset{´}{r})\delta (t-\overset{´}{t})$$

where $k_{B}$ is the Boltzmann constant, $\mu_{0}$ is the vacuum permeability, and V is cell volume [42].

The dimensions of the stepped nanowire used in this study are length (l), width (*w*), and thickness (*th*), with the values (*l* × *w* × *th*) = (1000 nm × 200 nm × *th*). The stepped area dimensions, depth (*d*) and length (*λ*), are (*d* nm × *λ* nm) as shown in Figure 1. The magnetic properties of in-plane magnetic materials, such as permalloy, were employed in this study [43]. A cubic mesh with a unit cell size smaller than the exchange lengths (*l_ex_* = 5.3 nm) is necessary, so a unit cell size of 5 nm was used in all simulations.

## 3. Results and Discussion

In this research, we simulate an innovative 3D model of a storage memory where the implementation of functional VDW pinning facilitates the writing and storing of information within a single stepped nanowire. However, the VDW pinning in the stepped nanowire is influenced by device temperature. Consequently, we conducted simulations under varying device temperatures to examine the thermal stability of the VDW structure both during its motion and within the stepped area during the pinning and depinning processes. The simulations utilized current density to drive the VDW in the stepped nanowires. Figure 1 depicts the dimensions of the stepped nanowire used to explore the thermal stability of the VDW. The VDW is nucleated at an appropriate current density, moving from left to right toward the stepped area.

This study investigates the VDW thermal stability by focusing on three key aspects: (1) the thermal transformation of VDW during its dynamic motion, (2) the influence of device temperature on VDW dynamics, and (3) the process of thermal pinning and depinning of VDW in the stepped area.

### 3.1. VDW Thermal Transformation

The steady motion of VDW in magnetic nanowires is described by the Thiele equation.

(3) $$F+G\times \left(v-u\right)+\overset{⃡}{D}\left(\alpha v-\beta u\right)=0$$

where F is the static force, G is the gyrovector (along the z-axis), and $\overset{⃡}{D}$ is the dissipation dyadic.

The VDW dynamics, as elucidated by this equation, is a consequence of the equilibrium between the restoring force, which confines the VDM within the wire, and the gyrovector, which induces the magnetization to circulate around the vortex core. However, parameters like the strength of the applied field, variations in device temperature, and the geometry of the wire may disrupt the balance, resulting in the transformation of the VDW into a TDW [4,44,45,46,47].

**Figure 2:**
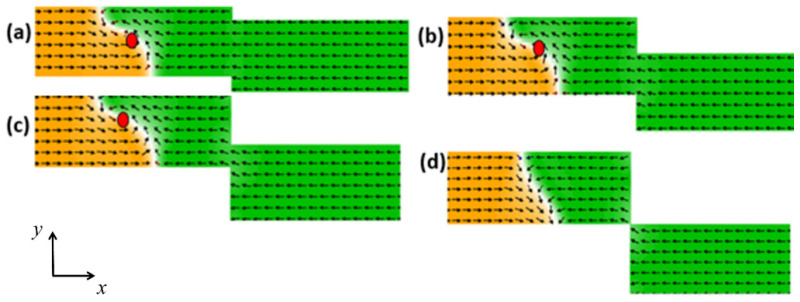
VDW dynamics show high structural stability in the stepped nanowire with pinning area dimensions of (**a**) (50 × 0 nm^2^), (**b**) (100 × 0 nm^2^), and (**c**) (150 × 0 nm^2^). (**d**) VDW transformation in the nanowire with stepped area dimensions of (200 × 0 nm^2^).

Therefore, the structural stability of the VDW during its dynamics in stepped nanowires was first investigated. The VDW was driven by a current density of 7.5 × 10^11^ Am^−2^ at a device temperature of 0 K, using different stepped nanowire pinning area sizes with overall nanowire dimensions of 1000 × 200 × 50 nm^3^. It was found that the VDW exhibits higher structural stability in nanowires with area dimensions of (50 × 0 nm^2^) [Figure 2a], (100 × 0 nm^2^) [Figure 2b], and (150 × 0 nm^2^) [Figure 2c] due to lower easy anisotropy along the y-axis and higher exchange energy, which keeps the DW magnetization curling around the VDW core and maintains the balance of restoring force and the gyrovector. However, when the depth of the stepped area was increased to (200 × 0 nm^2^) [Figure 2d], it was observed that the VDW lost the balance between restoring force and the gyrovector; as a result, it converted into a TDW after 2.5 ns of motion, attributed to the increased easy anisotropy along the y-axis with the deeper stepped area.

**Figure 3:**
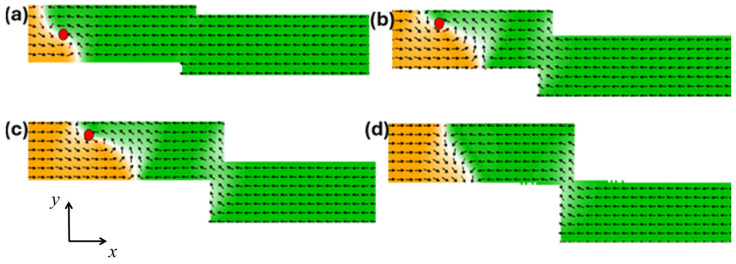
VDW dynamics with high structural stability in the stepped nanowire with pinning area dimensions of (**a**) (50 × 50 nm^2^), (**b**) (100 × 50 nm^2^), and (**c**) (150 × 50 nm^2^). (**d**) VDW transformation in the nanowire with stepped area dimensions of (200 × 50 nm^2^).

The same behavior of VDW dynamics in different structures [Figure 2] was observed when the stepped length was increased to *λ* = 50 nm, as shown in Figure 3.

Thus, in this study, nanowires with *λ* = 0 were used to investigate VDW transformation, while those with *λ* = 50 were used to investigate VDW dynamics and pinning. Increasing the step length helps to maintain the VDW structure during the pinning process, as reported in [48].

**Figure 4:**
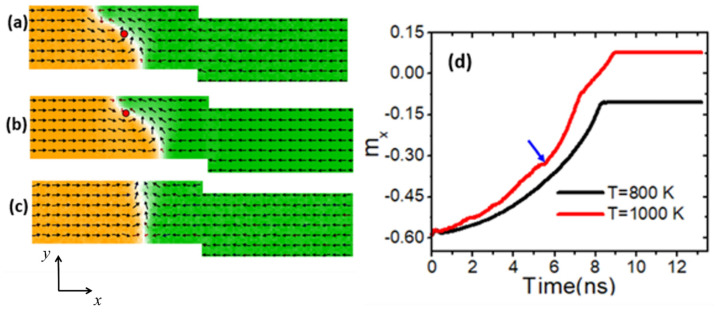
(**a**) VDW dynamics show high structural stability in the stepped nanowire with pinning area dimensions of (50 × 0 nm^2^). (**b**) The VDW reached the stepped area with high structural stability at a device temperature of 800 K. (**c**) The VDW transformation into TDW at a temperature of 1000 K. (**d**) *m_x_* versus time for two curves with two temperature (*T*) values.

As the device temperature increases, it is observed that the VDW core rises toward the edge until its core disappears, resulting in the transformation of the VDW into a TDW. Figure 4a illustrates the dynamics of the VDW in a nanowire with step (50 × 0 nm^2^) at a temperature of 400 K, where the VDW moves without transforming until it reaches the stepped region. However, at an elevated temperature of 800 K, the VDW core begins to rise, while the wall maintains greater structural stability as it approaches the stepped region, as shown in Figure 4b. Upon further increasing the temperature to 1000 K, the VDW core ascends until it reaches the nanowire edge, loses its structural stability, and transforms into a TDW, as depicted in Figure 4c. Figure 4d presents two graphs of *m_x_* versus time at device temperatures of 800 K and 1000 K. The black graph represents VDW dynamics at 800 K, showing a smooth trend without any sign of transformation. In contrast, the red graph represents VDW motion at 1000 K, indicating a transformation after 5 ns from the beginning of the motion (pointed to by the blue arrow).

**Figure 5:**
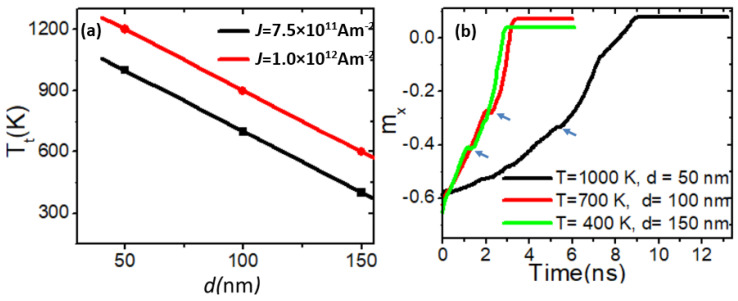
(**a**) *T_t_* as a function of *d* for two current density values. (**b**) *m_x_* versus time for three curves with different *d* values and at *T_t_*.

The same investigation was conducted with stepped area dimensions of 100 × 0 nm^2^ and 150 × 0 nm^2^. Figure 5a presents a graph of the transformation temperature (*T_t_*) as a function of stepped area depth (50 nm, 100 nm, and 150 nm) for two different current density values (*J* = 7.5 × 10^11^ Am^−2^ and 1.0 × 10^12^ Am^−2^). Figure 5b illustrates the relationship between *m_x_* and time for VDW motion in three nanowires with step depths of 50 nm, 100 nm, and 150 nm, each at its respective VDW transformation temperatures. The thermal stability of the VDW structure was found to decrease as the step depth (*d*) increased. For example, under a current density of *J* = $7.5\times 10^{11}\;Am^{-2}$, the VDW remained stable for approximately 5 ns (shown by the blue arrows) for a step depth of 50 nm, while for step depths of 100 nm and 150 nm, it remained stable for about 2 ns and 1 ns, respectively. The reduction in VDW stability time with increasing *d* is attributed to the rise in easy-axis anisotropy energy along the y-axis.

**Figure 6:**
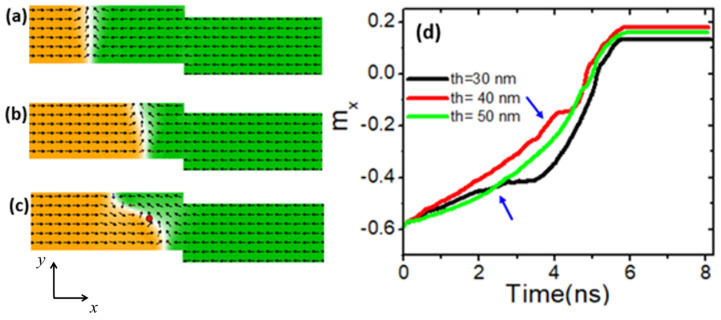
(**a**) VDW transformation with a device thickness of 30 nm and (**b**) 40 nm. (**c**) VDW with higher structural stability with a device thickness of 50 nm. (**d**) *m_x_* as a function of time for three different device thickness values and the device temperature of 600 K.

The thermal stability of the VDW structure can be further enhanced by adjusting the nanowire dimensions, such as width and thickness. In this study, we investigated the impact of increasing nanowire thickness on the thermal stability of the VDW structure. We began by varying the thickness of nanowires with stepped dimensions of 50 × 0 nm^2^ at a device temperature of 600 K. At this temperature, the VDW transformed into a TDW for thickness values below 50 nm, while the VDW exhibited greater thermal stability for thicknesses of 50 nm or more. Figure 6a illustrates the VDW transforming into a TDW at a thickness of 30 nm, and Figure 6b shows the transformation at 40 nm. However, when the thickness was increased to 50 nm, the VDW moved to the stepped area with enhanced structural stability, as depicted in Figure 6c. To better understand the effect of thickness on VDW thermal transformation, Figure 6d presents the variation of *m_x_* over time for three different nanowire thicknesses. It is evident that increasing the nanowire thickness enhances the VDW stability time. For instance, at a thickness of 30 nm, the VDW remained stable for approximately 2.5 ns (indicated by the blue arrow). With an increased thickness of 40 nm, the stability duration extended to 4 ns.

**Figure 7:**
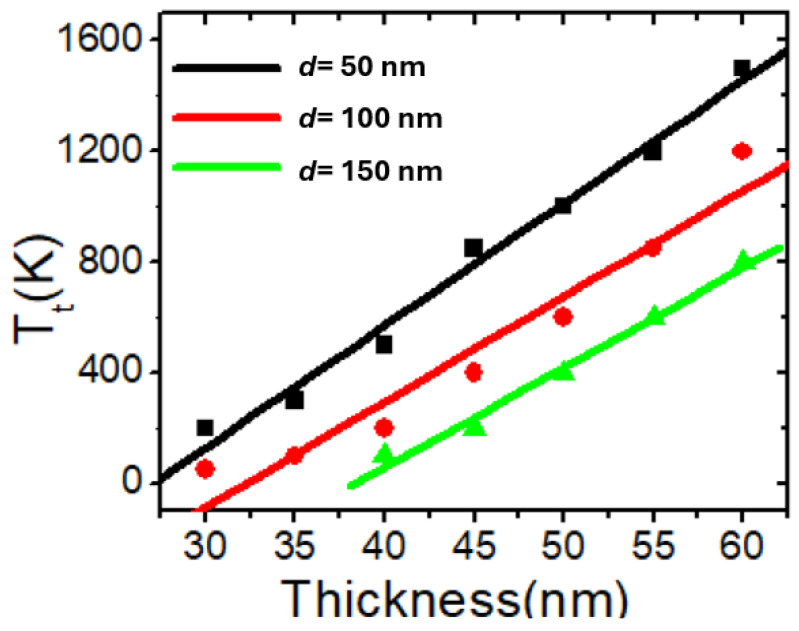
*T_t_* as a function of device thickness for nanowires with stepped areas of 50 × 0 nm^2^, 100 × 0 nm^2^, and 150 × 0 nm^2^.

A similar investigation into thermal VDW transformation was conducted by varying the device thickness using nanowires with stepped areas of 100 × 0 nm^2^ and 150 × 0 nm^2^. Figure 7 illustrates the relationship between *T_t_* and device thickness for these structures. It is observed that *T_t_* exhibits a linear relationship with respect to device thickness across the different structures.

### 3.2. VDW Thermal Dynamics

**Figure 8:**
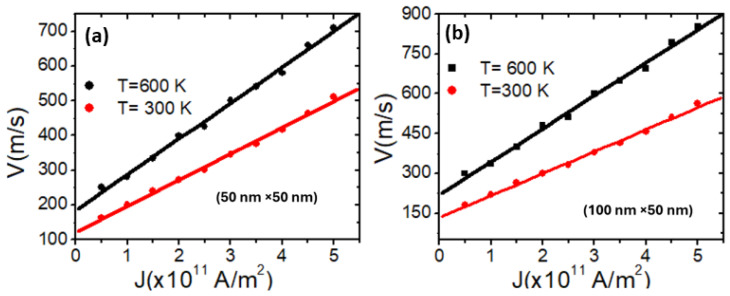
VDW velocity as a function of the current density for nanowires under device temperatures of 300 K and 600 K in the stepped nanowire with dimensions of (**a**) 50 × 50 nm^2^ and (**b**) 100 × 50 nm^2^.

This study explored the impact of device temperature on VDW velocity in two stepped nanowires with pinning area dimensions of 50 × 50 nm^2^ and 100 × 50 nm^2^. The results reveal that VDW velocity increases linearly with both current density and device temperature for both structures. Additionally, an increase in VDW velocity was observed with an increase in dimension *d*, attributed to the transformation of VDW into TDW, which moves faster than VDW. Figure 8a displays the relationship between VDW velocity and current density for the 50 × 50 nm^2^ structure at 300 K and 600 K, while Figure 8b illustrates the same relationship for the 100 × 50 nm^2^ structure. The relation between the VDW velocity and the current density can be described by the equation

(4) $$v=\frac{gP\beta \mu_{b}}{2e\alpha M_{s}}J$$

where *g* is the Lande factor, *P* is the spin polarization, β is the nonadiabatic parameter, $\mu_{b}$ is the Bohr magnetron, J is the current density, *e* is the carrier charge, α is the Gilbert damping factor, and $M_{s}$ is saturation magnetization [4,44].

### 3.3. VDW Thermal Pinning and Depinning

**Figure 9:**
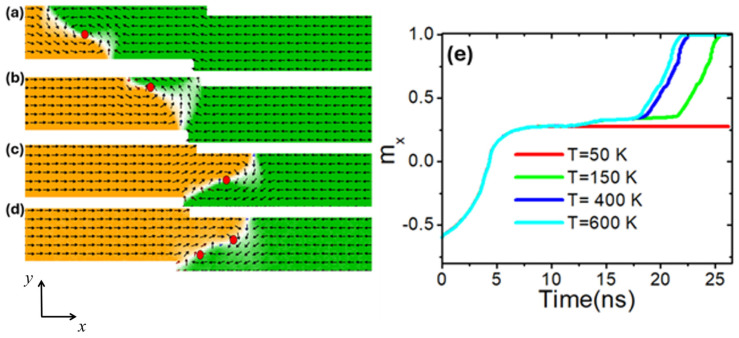
(**a**) VDW dynamics in a stepped nanowire with stepped dimensions of 50 × 50 nm^2^. (**b**) VDW pinning in the stepped area at a device temperature of 50 K. (**c**) VDW depinning from the stepped area at the device temperature of 150 K. (**d**) VDW depinning at a device temperature of 600 K with two vortices. (**e**) Magnetization components point along the *x*-direction (*m_x_*) as a function of time at different device temperatures.

Further investigations focused on the thermal pinning and depinning of the VDW as it reached the stepped area. In this study, a device thickness of 60 nm and *λ* = 50 were employed to ensure that the VDW approached the stepped area with optimal structural stability. In the stepped area, we examined thermal pinning using nanowire dimensions of 50 × 50 nm^2^. Initially, the VDW was driven by current density toward the stepped area [Figure 9a] to pin it there [Figure 9b]. Subsequently, the device temperature was then increased until the VDW depinned from the area to the edge of the nanowire. The depinning process was examined under various temperatures. At a current density of 7.5 × 10^11^ Am^−2^ and a device temperature of 50 K, the VDW was initially pinned in the stepped area, as shown in Figure 9b and the red graph depicting the relation of normalized magnetization (*m_x_*) versus time in Figure 9e. By increasing the temperature to below 150 K, the VDW remained pinned in the stepped area. However, at temperatures of 150 K or higher, VDW thermal pinning decreased, leading to the depinning [Figure 9c,e, green graph]. Higher device temperatures resulted in reduced pinning durations for the VDW. For instance, the VDW remained pinned for 22 ns at 150 K, 18 ns at 400 K, and 15 ns at 600 K. During thermal depinning, the VDW changed its chirality to a clockwise direction. Additionally, at temperatures exceeding 400 K, the VDW exhibited two vortices after depinning from the stepped area [Figure 9d]. The rise in device temperature resulted in greater spin fluctuations and VDW oscillations, which may facilitate the release of the VDW from the stepped region [49].

**Figure 10:**
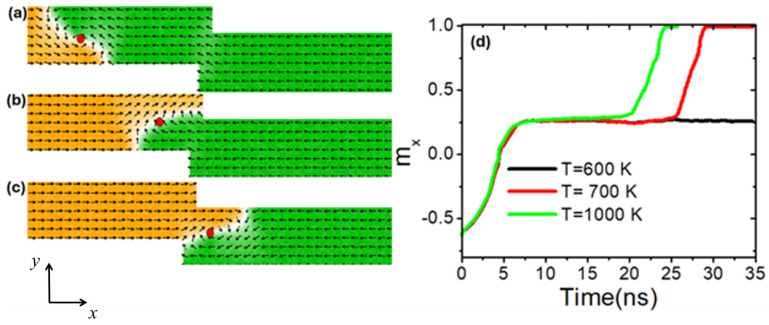
(**a**) VDW dynamics in a stepped nanowire with stepped dimensions of 100 × 50 nm^2^. (**b**) VDW pinning in the stepped area at a device temperature of 600 K. (**c**) VDW depinning from the stepped area at a device temperature of 700 K. (**d**) Magnetization components pointing along the *x*-direction (*m_x_*) as a function of time at different device temperatures (600 K, 700 K, and 1000 K).

To better understand the effects of stepped region dimensions on VDW thermal pinning, a nanowire with stepped area dimensions of 100 × 50 nm^2^ was utilized. The VDW dynamics as it approached the constricted area are depicted in Figure 10a, while Figure 10b illustrates VDW pinning at the pinning area. It was discovered that the VDW exhibits high thermal pinning at temperatures below 600 K, as shown in Figure 10b and the black graph for 600 K in Figure 10d. However, at a device temperature of 700 K, VDW remained pinned for 26 ns before depinning from the stepped area, as shown in Figure 10c and the red graph in Figure 10d. When the device temperature increased to 1000 K, VDW stayed for 20 ns before depinning from the stepped area, as shown in Figure 10c and the green graph in Figure 10d. After VDW depinning from the stepped area, similar behavior was observed as with the stepped area dimensions of 50 × 50 nm^2^, where VDW changed its chirality to clockwise. However, with stepped dimensions of 100 × 50 nm^2^, VDW exhibited more thermal stability after moving away from the pinning area and did not show VDW with two vertices at high temperatures.

**Figure 11:**
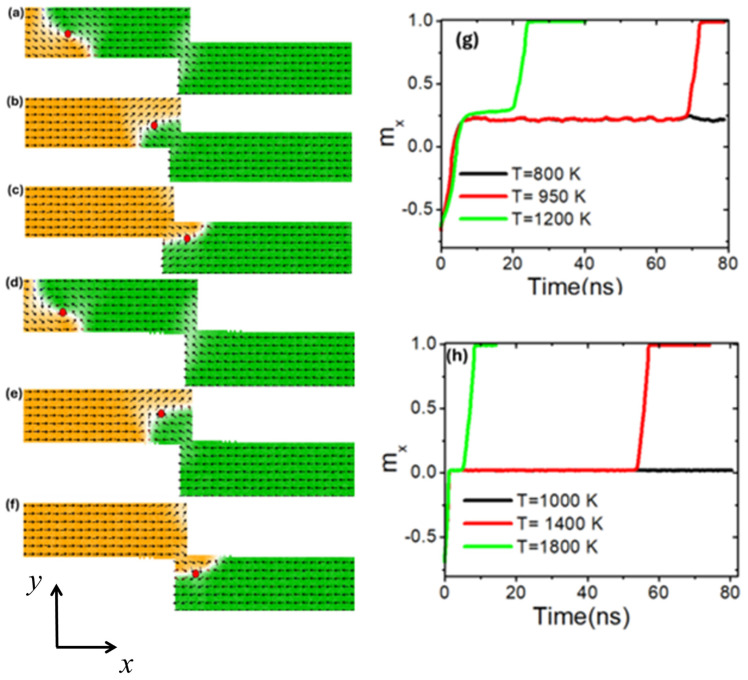
(**a**) VDW dynamics in a stepped nanowire with stepped dimensions of 150 × 50 nm^2^. (**b**) VDW pinning in the stepped area at a device temperature of 800 K. (**c**) VDW depinning from the stepped area at a device temperature of 950 K. (**d**) VDW dynamics in stepped nanowire with stepped dimensions of 200 × 50 nm^2^. (**e**) VDW pinning in the stepped area at a device temperature of 1000 K. (**f**) VDW depinning in the stepped area at the device temperature of 1400 K. (**g**) Magnetization components along the x-direction (*m_x_*) over time at temperatures of 800 K, 1000 K, and 1200 K in a stepped nanowire of 150 × 50 nm^2^. (**h**) Magnetization components along the *x*-direction (*m_x_*) over time at temperatures of 1000 K, 1400 K, and 1800 K in a stepped nanowire of 200 × 50 nm^2^.

To achieve higher thermal pinning in the stepped area, a nanowire with pinning area dimensions of 150 × 50 nm^2^ was utilized. The results demonstrate that the VDW exhibits significant resistance to depinning in the stepped area as the device temperature increases. The VDW began depinning from the stepped area at a device temperature of 950 K [Figure 11c,g, red graph], remaining pinned for approximately 70 ns before moving away. When the device temperature was increased to 1200 K, the VDW stayed pinned for around 20 ns before depinning from the stepped area to the end of the nanowire as shown in Figure 11g, green graph.

Increased thermal stability of the VDW was observed at higher device temperatures in stepped nanowires with constricted dimensions of 200 × 50 nm^2^. Figure 11d shows the VDW movement within a stepped nanowire of these dimensions, while Figure 11e,f illustrate VDW pinning and depinning. The data indicate that the VDW remained pinned until device temperatures reached 1400 K, as evidenced by Figure 11h (red plot), which shows VDW staying for 50 ns before leaving the stepped area and moving to the end of the nanowire.

**Figure 12:**
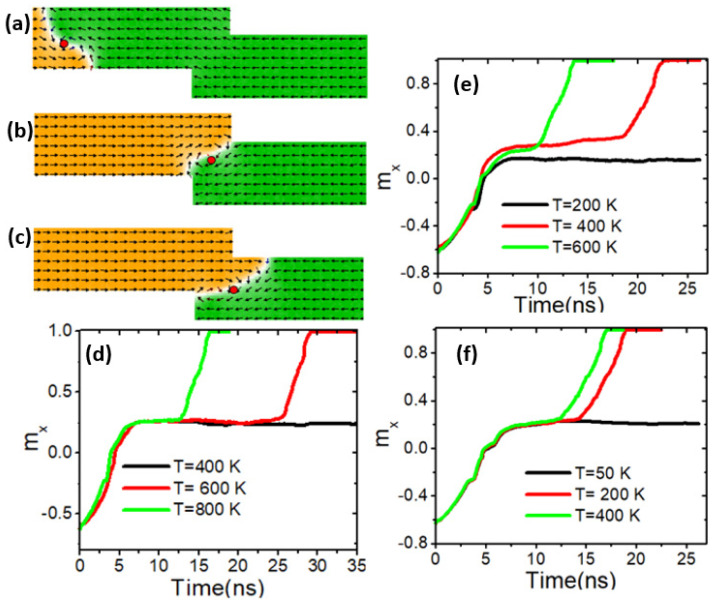
(**a**) VDW dynamics in stepped nanowire with stepped dimensions of 100 × 100 nm^2^. (**b**) VDW pinning in the stepped area (100 × 100 nm^2^) at a device temperature of 400 K. (**c**) VDW depinning from the stepped area (100 × 100 nm^2^) at the device temperature of 600 K. (**d**) The analysis of the magnetization components along the x-direction (*m_x_*) over time at temperatures of 400 K, 600 K, and 800 K in a stepped nanowire of 100 × 100 nm^2^. (**e**) Magnetization components along the *x*-direction (*m_x_*) over time at temperatures of 200 K, 400 K, and 600 K in a stepped nanowire of 100 × 150 nm^2^. (**f**) Magnetization components along the *x*-direction (*m_x_*) over time at temperatures of 50 K, 200 K, and 400 K in a stepped nanowire of 100 × 200 nm^2^.

The length of the step (*λ*) is another dimension that affects the thermal VDW pinning and depinning. In this analysis, the step length was varied in 50 nm increments. Figure 12a depicts VDW dynamics in a stepped nanowire with dimensions of 100 nm × 100 nm. Figure 12b shows VDW pinning at these dimensions and a device temperature of 400 K. It was found that the VDW depinned from this step size starting at 600 K, as illustrated in Figure 12c and the red graph in Figure 12d. When the step length was increased to 150 nm (100 nm × 150 nm), thermal VDW depinning occurred at 400 K (Figure 12e), and at 200 K with a step size of 100 nm × 200 nm (Figure 12f). These results indicate that VDW thermal depinning increases with step length, allowing the VDW to leave the pinning area at lower temperatures.

**Figure 13:**
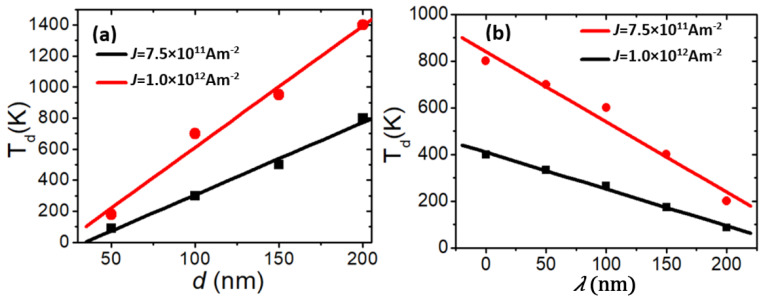
(**a**) The plot of *T_d_* as a function of *d* for two values of current density and λ = 50 nm. (**b**) The plot of *T_d_* versus *λ* and *d* = 100 nm.

Here is a summary of how the step depth and length affect VDW thermal stability. Figure 13a shows the relationship between depinning temperature (*T_d_*) and depth (*d*) for two current density values. The data reveal that *T_d_* has a linear relationship with *d*, increasing as *d* increases. Conversely, Figure 13b illustrates that *T_d_* decreases as the step length (*λ*) increases.

**Figure 14:**
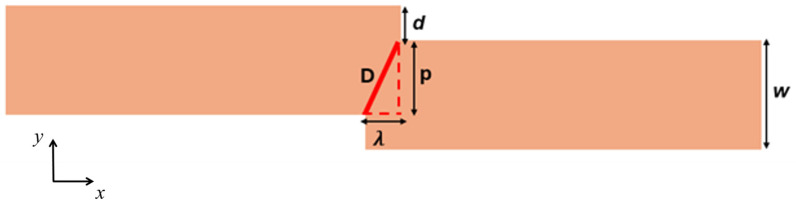
The stepped area diagonal (D) with dimensions of *λ* and p, where $D^{2}=\lambda^{2}+p^{2}$.

The thermal VDW pinning strength can be analytically described by the diagonal length (D) of the stepped area. The diagonal length of the stepped area is given by the equation

(5) $$D^{2}=\lambda^{2}+p^{2}$$

where p=w−d, w is the nanowire width, *λ* is the stepped area length, and *d* is the stepped area depth, as shown in Figure 14.

According to this equation, increasing *d* results in a reduction in D, thereby enhancing thermal pinning strength. Similar effects are obtained when λ is decreased.

**Figure 15:**
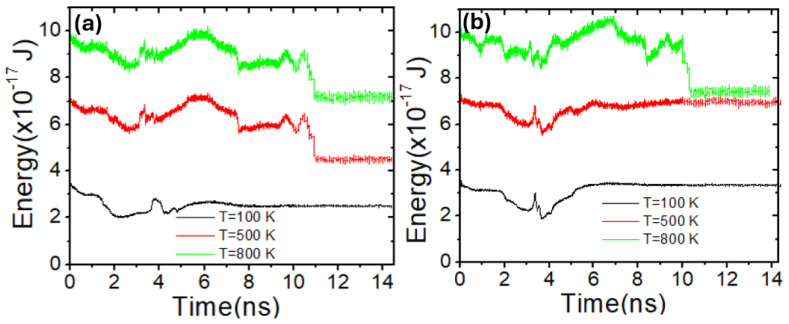
(**a**) VDW energy as a function of time for three device temperatures with a stepped area of 50 × 50 nm^2^. (**b**) VDW energy versus time for three values of device temperatures with a stepped area of 100 × 50 nm^2^.

To further confirm that the VDW is more likely to depin from the stepped area as the device temperature increases, we investigated VDW energy by varying device temperature in two structures with stepped areas of 50 × 50 nm^2^ and 100 × 50 nm^2^. Figure 15a shows the VDW energy over time for three different device temperatures in a stepped area with dimensions of 50 × 50 nm^2^. It was found that VDW energy increases as the device temperature rises. Moreover, it was observed that VDW energy decreases from approximately 9.0 × 10^−17^ J to around 7.0 × 10^−17^ J after 10 ns during the depinning process in the stepped area at a device temperature of 800 K, as indicated by the green curve. In contrast, at temperatures of 100 K (black curve) and 500 K (red curve), there is a small reduction in the VDW energy when it reaches the stepped area after 4 ns, after which the VDW becomes completely pinned and begins to gain energy. On the other hand, Figure 15b illustrates the VDW energy over time for the same values of device temperature (100 K, 500 K, and 800 K) with stepped area dimensions of 100 × 50 nm^2^. It was noted that at temperatures of 500 K and 800 K, VDW energy decreases during the depinning process after 11 ns. However, at 100 K, a reduction in energy was observed only during the pinning process.

## 4. Conclusions

In this study, we investigated the thermal pinning and depinning behaviors of vortex domain walls (VDWs) within stepped magnetic nanowires, emphasizing their implications for storage memory nanodevices. Our findings highlight the critical role of thermal fluctuations in modulating the stability and mobility of VDWs, thereby influencing the efficiency and reliability of memory devices that utilize these nanostructures. Through comprehensive simulations, we established that the thermal activation energy required for depinning VDWs from constrictions is significantly influenced by the geometric parameters of the nanowires and the temperature. Specifically, narrower constrictions and lower temperatures were found to increase the pinning strength, thereby enhancing the stability of the stored magnetic states. Conversely, at higher temperatures, the depinning probability increases, potentially leading to undesired switching events. These insights into the thermal dynamics of VDWs in magnetic nanowires furnish critical guidelines for optimizing the design and operation of next-generation storage memory devices. By carefully engineering the dimensions of the constricted regions and controlling the operational temperature, it is possible to achieve a balance between stability and switch ability, crucial for the performance of high-density, thermally robust memory technologies. Overall, our study underscores the importance of considering thermal effects in the development of magnetic nanowire-based memory devices. Future research should focus on exploring additional material systems and nanowire geometries to further advance the understanding and application of VDWs in practical memory storage solutions.

## Data Availability

Data are contained within the article.

## References

[1] Parkin S.S., Hayashi M., Thomas L. (2008). Magnetic domain-wall racetrack memory. Science.

[2] Hayashi M., Thomas L., Moriya R., Rettner C., Parkin S.S. (2008). Current-controlled magnetic domain-wall nanowire shift register. Science.

[3] Allwood D.A., Xiong G., Faulkner C.C., Atkinson D.P.D., Cowburn R.P. (2005). Magnetic domain-wall logic. Science.

[4] Thiaville A., Nakatani Y., Miltat J., Suzuki Y. (2005). Micromagnetic understanding of current-driven domain wall motion in patterned nanowires. Europhys. Lett..

[5] Hayashi M., Thomas L., Moriya R., Rettner C., Parkin S.S.P. (2008). Current-driven domain wall veloci-ties exceeding the spin angular momentum transfer rate in permalloy nanowires. Phys. Rev. Lett..

[6] Tveten E.G., Qaiumzadeh A., Tretiakov O.A., Brataas A. (2013). Staggered dynamics in antiferromagnets by collective coordinates. Phys. Rev. Lett..

[7] Shiino T., Oh S., Haney P.M., Lee H.-W., Go G., Park B.-G., Lee K.-J. (2016). Antiferromagnetic domain wall motion driven by spin-orbit torques. Phys. Rev. Lett..

[8] Sampaio J., Cros V., Rohart S., Thiaville A., Fert A. (2013). Nucleation, stability and current-induced motion of isolated magnetic skyrmions in nanostructures. Nat. Nanotechnol..

[9] Tomasello R., Martinez E., Zivieri R., Torres L., Carpentieri M., Finocchio G. (2014). A strategy for the design of skyrmion racetrack memories. Sci. Rep..

[10] Meier G., Bolte M., Eiselt R., Krüger B., Kim D.H., Fischer P. (2007). Direct imaging of stochastic domain-wall motion driven by nanosecond current pulses. Phys. Rev. Lett..

[11] Nakatani Y., Thiaville A., Miltat J. (2005). Head-to-head domain walls in soft nano-strips: A refined phase dia-gram. J. Magn. Magn. Mater..

[12] Beach G.S., Nistor C., Knutson C., Tsoi M., Erskine J.L. (2005). Dynamics of field-driven domain-wall propaga-tion in ferromagnetic nanowires. Nat. Mater..

[13] Yamaguchi A., Ono T., Nasu S.M.K., Mibu K., Shinjo T. (2004). Real-space observation of current-driven domain wall motion in submicron magnetic wires. Phys. Rev. Lett..

[14] Kläui M., Jubert P.O., Allenspach R., Bischof A., Bland J.A.C., Faini G., Vouille C. (2005). Direct observation of domain-wall configurations transformed by spin currents. Phys. Rev. Lett..

[15] Ono T., Miyajima H., Shigeto K., Mibu K., Hosoito N., Shinjo T. (1999). Propagation of a magnetic domain wall in a submicrometer magnetic wire. Science.

[16] Yang S.H., Ryu K.S., Parkin S. (2015). Domain-wall velocities of up to 750 ms^−1^ driven by exchange-coupling torque in synthetic antiferromagnets. Nat. Nanotechnol..

[17] Slonczewski J.C. (1996). Current-driven excitation of magnetic multilayers. J. Magn. Magn. Mater..

[18] Berger L. (1996). Emission of spin waves by a magnetic multilayer traversed by a current. Phys. Rev. B.

[19] Yuan H.Y., Wang X.R. (2014). Domain wall pinning in notched nanowires. Phys. Rev. B.

[20] Kurniawan C., Djuhana D. (2016). Current driven domain wall depinning in notched Permalloy nanowires. AIP Conference Proceedings.

[21] Brandão J., Novak R.L., Lozano H., Soledade P.R., Mello A., Garcia F., Sampaio L.C. (2014). Control of the mag-netic vortex chirality in Permalloy nanowires with asymmetric notches. J. Appl. Phys..

[22] Djuhana D., Kurniawan C., Kim D.H. (2018). Micromagnetic study of domain wall depinning driven by nano-second current pulse in notched Permalloy nanowires. Curr. Appl. Phys..

[23] Shiu D.S., Hong Y., Su C.H., Lai K.F., Wu J.C., Lin L., Horng L. (2019). Depinning field of vortex domain wall in wide magnetic wires with asymmetric notches using magneto-optical kerr effect microscopy. J. Elec-Tronic Mater..

[24] Kurniawan C., Soegijono B., Djuhana D. (2019). Investigation of notch dept effect on domain wall depinning in ferromagnetic nanowires by micromagnetic simulation. IOP Mater. Sci. Eng..

[25] Al Bahri M. (2022). Controlling domain wall thermal stability switching in magnetic nanowires for storage memory nanodevices. J. Magn. Magn. Mater..

[26] Al Bahri M., Al Harthy T. (2023). Vortex Domain Wall Thermal Stability in Magnetic Nanodevices with In-Plane Magnetic Anisotropy. Phys. Status Solidi A.

[27] Seo S.M., Kim K.W., Ryu J., Lee H.W., Lee K.J. (2012). Current-induced motion of a transverse magnetic domain wall in the presence of spin Hall effect. Appl. Phys. Lett..

[28] Ai J.H., Miao B.F., Sun L., You B., Hu A., Ding H.F. (2011). Current-induced domain wall motion in permalloy nanowires with a rectangular cross-section. J. Appl. Phys..

[29] Wieser R., Nowak U., Usadel K.D. (2004). Domain wall mobility in nanowires: Transverse versus vortex wall. Phys. Rev. B.

[30] Malinowski G., Longa F.D., Rietjens J.H.H., Paluskar P.V., Huijink R., Swagten H.J.M., Koopmans B. (2008). Control of speed and efficiency of ultrafast demagnetization by direct transfer of spin angular momentum. Nat. Phys..

[31] Al Bahri M. (2021). Geometrical Confinement of Vortex Domain Wall in Constricted Magnetic Nanowire with In-Plane Magnetic Anisotropy. IEEE Trans. Magn..

[32] Zhang S., Zhang S.S.L. (2009). Generalization of the Landau-Lifshitz-Gilbert equation for conducting ferro-magnets. Phys. Rev. Lett..

[33] Tatara G., Kohno H. (2004). Theory of current-driven domain wall motion: Spin transfer versus momentum transfer. Phys. Rev. Lett..

[34] Schieback C., Hinzke D., Kläui M., Nowak U., Nielaba P. (2009). Temperature dependence of the current-induced domain wall motion from a modified Landau-Lifshitz-Bloch equation. Phys. Rev. B—Condens. Matter Mater. Phys..

[35] Brataas A., Bauer G.E., Kelly P.J. (2006). Non-collinear magnetoelectronics. Phys. Rep..

[36] Kent A.D., Rüdiger U., Yu J., Thomas L., Parkin S.S. (1999). Magnetoresistance, micromagnetism, and domain wall effects in epitaxial Fe and Co structures with stripe domains. J. Appl. Phys..

[37] Al Bahri M. (2020). Vortex domain wall dynamics in stepped magnetic nanowire with in-plane magnetic anisotropy. J. Magn. Magn. Mater..

[38] Cowburn R.P., Koltsov D.K., Adeyeye A.O., Welland M.E., Tricker D.M. (1999). Single-domain circular nano-magnets. Phys. Rev. Lett..

[39] Al Bahri M., Borie B., Jin T.L., Sbiaa R., Kläui M., Piramanayagam S.N. (2019). Staggered magnetic nanowire de-vices for effective domain-wall pinning in racetrack memory. Phys. Rev. Appl..

[40] Donahue M., Porter D.G. (1999). OOMMF User’s Guide, Version 1.0.

[41] Zhang S., Li Z. (2004). Roles of Nonequilibrium Conduction Electrons on the Magnetization Dynamics of Ferro-magnets. Phys. Rev. Lett..

[42] Martinez E., Lopez-Diaz L., Torres L., Tristan C., Alejos O. (2007). Thermal effects in domain wall motion: Micromagnetic simulations and analytical model. Phys. Rev. B—Condens. Matter Mater. Phys..

[43] Bahri A., Al-Kamiyani S. (2024). Thermal Effects on Domain Wall Stability at Magnetic Stepped Nanowire for Nanodevices Storage. Nanomaterials.

[44] Thiele A. (1973). Steady-State Motion of Magnetic Domains. Phys. Rev. Lett..

[45] Ye C., Li L., Shu Y., Li Q., Xia J., Hou Z., Zhou Y., Liu X., Yang Y., Zhao G. (2022). Generation and manipulation of skyrmions and other topological spin structures with rare metals. Rare Met..

[46] Shen L., Xia J., Zhang X., Ezawa M., Tretiakov O.A., Liu X., Zhou Y. (2020). Current-Induced Dynamics and Chaos of Antiferromagnetic Bimerons. Phys. Rev. Lett..

[47] Zhang X., Zhou Y., Ezawa M. (2016). Magnetic bilayer-skyrmions without skyrmion Hall effect. Nat. Commun..

[48] Al Bahri M. (2022). Chiral Dependence of Vortex Domain Wall Structure in a Stepped Magnetic Nanowire. Phys. Status Solidi A.

[49] Sbiaa R., Bahri M.A., Piramanayagam S.N. (2018). Domain wall oscillation in magnetic nanowire with a geometri-cally confined region. J. Magn. Magn. Mater..

